# US Adults’ Perceptions of the Harmful Effects During Pregnancy of Using Electronic Vapor Products Versus Smoking Cigarettes, *Styles* Survey, 2015

**DOI:** 10.5888/pcd13.160349

**Published:** 2016-12-22

**Authors:** Kimberly H. Nguyen, Van T. Tong, Kristy L. Marynak, Brian A. King

**Affiliations:** 1 Office on Smoking and Health, National Center for Chronic Disease Prevention and Health Promotion, Centers for Disease Control and Prevention, Atlanta, Georgia; 2 Division of Reproductive Health, National Center for Chronic Disease Prevention and Health Promotion, Centers for Disease Control and Prevention, Atlanta, Georgia.

## Abstract

**Introduction:**

Research suggests aerosol from electronic vapor products (EVPs) has fewer harmful constituents than conventional cigarette smoke. Even so, EVPs and other nicotine-containing products are not safe to use during pregnancy. We examined perceptions among US adults regarding harm in using EVPs rather than smoking cigarettes during pregnancy.

**Methods:**

Data came from the 2015 *Styles* Survey, an Internet panel survey of a sample of US adults aged 18 years or older (N = 4,127). Perceived harm was assessed by asking respondents whether using EVPs was less, equally, or more harmful for pregnant women than smoking cigarettes. Descriptive statistics were used to estimate perceived harm overall and by sociodemographic characteristics and tobacco-use status. Perceived harm was assessed among all adults, women of reproductive age (18–44 years, n = 820), and women of nonreproductive age (≥45 years, n = 1,398).

**Results:**

Among all adults, 11.1% believed using EVPs during pregnancy was less harmful than smoking conventional cigarettes, 51.0% believed it was equally harmful, 11.6% believed it was more harmful, and 26.2% did not know. Prevalence of perception of less harm, by demographic category, was greatest among adults aged 18 to 24 years, men, non-Hispanic whites, adults with less than a high school diploma, current EVP users, and current cigarette smokers (*P* < .05). Prevalence of perception of less harm was greater among women of reproductive age (9.6%) than among those of nonreproductive age (7.9%) (*P* < .05).

**Conclusion:**

US adults have varying levels of perceptions about the harms of EVP use versus cigarette smoking during pregnancy. Efforts are warranted to prevent nicotine exposure during pregnancy and to educate adults on the dangers of using any form of tobacco during pregnancy, including EVPs.

## Introduction

Electronic vapor products (EVPs), including electronic cigarettes (e-cigarettes), are battery-powered devices that heat a liquid that typically contains nicotine and flavoring to produce an aerosol that is inhaled by the user (1–3). The proportion of women who use EVPs during pregnancy is unknown; however, an estimated 8% of pregnant women in the United States smoke cigarettes, exposing more than 320,000 fetuses annually to nicotine and other toxicants (4).

Although research indicates that EVP aerosol has fewer harmful constituents than cigarette smoke (4), EVPs and other nicotine-containing products are not safe to use during pregnancy (5,6). Nicotine exposure during pregnancy can contribute to increased risk of sudden infant death syndrome, low birthweight, preterm births, and adverse brain and lung development in fetuses and infants (3,7). Additionally, some flavorings used in EVPs can be toxic to human embryonic stem cells (8). Furthermore, although cessation from conventional cigarettes is a commonly cited reason for e-cigarette use, the current scientific evidence is insufficient to recommend use of EVPs for tobacco cessation among adults, including pregnant women (9).

In recent years, EVP use has increased among US youths and adults (10,11). National surveys indicate that female nonsmokers and smokers are more likely than their male counterparts to experiment with EVPs, and EVP use is increasing among women of reproductive age and among adults overall aged 25 to 44 years (10,11). Despite increased use of these products in recent years and the risks of use during pregnancy, little is known about public perceptions about the use of these products, particularly among pregnant women. Some studies found that EVPs were perceived as a safer alternative to smoking cigarettes, including during pregnancy (12–16). These studies were small in size and limited to specific populations such as a university-based outpatient clinic. Our study assessed perceptions of harm from EVP use versus cigarette smoking during pregnancy by using a nationally representative survey of US adults.

## Methods

Data came from *Styles*, a series of seasonal national consumer surveys conducted by Porter Novelli (10,11). *Styles* draws from KnowledgePanel*,* an Internet panel recruited by using probability-based sampling to reach respondents regardless of landline phone or Internet access. *Styles* is conducted among a nationally representative sample of US adults aged 18 years or older. Probability-based, stratified, random sampling is used to select panel respondents. *Styles* was fielded June 11–29, 2015, and 4,127 participants completed the survey, yielding a 67% response rate. Data were weighted to 2014 Current Population Survey distributions by sex, age, annual household income, race/ethnicity, household size, education, US census region, metropolitan status, and Internet access (17). Our analysis of *Styles* data was deemed exempt from institutional review board approval because it was a secondary analysis of de-identified data.

Perception of harm was determined by asking the question “Do you believe it is less harmful, equally harmful, or more harmful for pregnant women to use electronic vapor products than to smoke regular cigarettes?” Response options were less harmful, equally harmful, more harmful, and don’t know.

*Current cigarette smokers* were defined as respondents who reported smoking at least 100 cigarettes during their lifetime and who smoked cigarettes every day or some days at the time of the survey. *Former smokers* were respondents who smoked at least 100 cigarettes during their lifetime, and who smoked not at all at the time of survey. *Never smokers* were respondents who smoked fewer than 100 cigarettes in their lifetime.

Participants were asked “Have you ever tried any of the following products, even just one time?”; to answer, respondents selected from a list of products those they had ever used and those they had used during the previous 30 days (“In the past 30 days, which of the following products have you used at least once?”). EVP use was defined on the basis of 3 response options: 1) used e-cigarettes, such as Blu, 21^st^ Century Smoke, or NJOY; 2) used electronic hookahs, hookah pens, or vape pens, such as Starbuzz or Fantasia; or 3) used other EVPs such as electronic cigars or electronic pipes. Current EVP users were respondents who reported ever using EVPs, even once, and who used an EVP in the previous 30 days. Former EVP users were those who reported ever using EVPs, even once, but who had not used EVPs in the previous 30 days. Never EVP users were those who reported never using an EVP.

Characteristics evaluated were sex, age, race/ethnicity, education, annual household income, marital status, US census region, and whether one or more children aged younger than 18 years lived in the household.

Percentages and 95% confidence intervals were computed to assess harm perceptions about EVP versus conventional cigarette use by pregnant women among 3 groups: 1) all adults, 2) women of reproductive-age (18–44 y), and 3) women of nonreproductive age (≥45 y). We performed χ^2^ tests to assess differences between harm perceptions and demographic characteristics and tobacco use (*P* < .05). Additionally, for each harm perception category, pairwise comparisons of proportions were computed to assess differences (*P* < .05) between women of reproductive and women of nonreproductive age for each sociodemographic group. Analyses were conducted using SAS version 9.3 (SAS Institute, Inc), and data were weighted to adjust for selection and nonresponse.

## Results

Of all respondents, 14.2% were current cigarette smokers, and 5.6% were current EVP users. Among all adults, perceptions of EVP use relative to cigarette smoking were as follows: less harmful (11.1%); equally harmful (51.0%); more harmful (11.6%); and don’t know (26.2%) (Table 1). By subpopulation, the perception that EVP use during pregnancy was less harmful than cigarette smoking was highest among males, adults aged 18–24 years, non-Hispanic whites, those with less than a high school diploma, those with an annual household income of $100,000 or more, those with children aged younger than 18 years living in the household, current cigarette smokers, and current EVP users.

**Table 1:** Perceptions About Harmful Effects of Electronic Vapor Products (EVPs) Versus Cigarette Smoking During Pregnancy^a^ Among Adults Aged 18 Years or Older (N = 4,127), by Sociodemographic Characteristics, *Styles* Survey, United States, 2015

Characteristic	All Adults aged ≥18 years			
	Less Harmful % (95%CI)	Equally Harmful % (95%CI)	More Harmful % (95%CI)	Do Not Know % (95%CI)
**Overall**	11.1 (10.0–12.3)	51.0 (49.2–52.8)	11.6 (10.4–12.8)	26.2 (24.6–27.8)
**Sex **				
Female	8.7 (7.2–10.1) **b**	54.7 (52.2–57.1)	11.5 (9.9–13.2)	25.1 (23.0–27.2)
Male	13.8 (12.0–15.6)	47.1 (44.5–49.8)	11.7 (9.9–13.5)	27.4 (25.0–29.7)
**Age**				
18–24	15.4 (10.7–20.0)^b^	52.3 (46.1–58.4)	10.3 (6.5–14.1)	22.1 (17.0–27.1)
25–44	10.7 (8.8–12.7)	53.9 (50.5–57.2)	10.9 (8.6–13.1)	24.5 (21.7–27.4)
45–64	11.8 (10.0–13.5)	48.7 (46.0–51.4)	11.6 (9.8–13.5)	27.9 (25.5–30.3)
≥ 65	7.9 (5.9–10.0)	49.5 (45.7–53.2)	13.9 (11.2–16.5)	28.7 (25.3–32.1)
**Race/ethnicity**				
Non-Hispanic white	12.7 (11.3–14.0)^b^	52.1 (50.0–54.1)	8.0 (6.9–9.1)	27.3 (25.5–29.1)
Non-Hispanic black	8.7 (5.6–11.8)	40.1 (34.6–45.5)	22.5 (18.0–27.5)	28.5 (23.4–33.6)
Hispanic	8.6 (5.3–11.8)	51.6 (46.2–56.9)	15.4 (11.5–19.3)	24.5 (19.3–29.0)
Non-Hispanic other	6.8 (2.9–10.7)	57.9 (49.7–66.0)	18.6 (12.0–25.2)	16.7 (10.6–22.9)
**Education**				
<High school diploma	12.9 (8.5–17.3) **b**	39.8 (33.6–12.3)	16.9 (12.2–21.6)	30.3 (24.4–36.3)
High school graduate	10.4 (8.5–12.3)	50.1 (46.9–53.4)	12.1 (9.8–14.3)	27.4 (24.6–30.2)
Some college	10.6 (8.6–12.7)	52.4 (49.1–55.6)	11.8 (9.7–14.0)	25.2 (22.4–28.0)
College degree or more	11.6 (9.7–13.6)	55.3 (52.2–58.4)	8.8 (6.9–10.7)	24.2 (21.6–26.9)
**Annual household income, $**				
<25,000	11.9 (9.1–14.7) **b**	40.9 (36.7–45.1)	15.4 (12.1–18.7)	31.8 (27.8–35.7)
25,000–49,999	8.1 (6.2–9.8)	49.0 (45.4–52.5)	14.9 (12.2–17.5)	28.1 (24.9–31.4)
50,000–99,999	10.9 (8.9–12.9)	53.1 (50.0–56.2)	10.2 (8.2–12.2)	25.8 (23.1–28.5)
≥100,000	13.7 (11.2–16.3)	57.4 (53.7–61.1)	7.9 (5.8–10.1)	20.9 (17.9–23.9)
**Marital status**				
Married/living with partner	11.1 (9.8–12.5)	52.0 (49.7–54.2)	11.1 (9.6–12.6)	25.8 (23.8–27.7)
Single	12.4 (9.8–15.1)	50.6 (46.6–54.5)	11.9 (9.3–14.5)	25.2 (21.8–28.5)
Divorced/widowed/separated	8.6 (6.2–10.9)	47.9 (43.4–52.3)	13.5 (10.3–16.6)	30.1 (25.9–34.3)
**US census region^c^**				
Northeast	11.8 (9.0–14.6)	49.4 (45.2–53.6)	10.0 (7.4–12.6)	28.8 (25.0–32.6)
Midwest	13.3 (10.9–15.8)	51.5 (48.0–55.0)	8.7 (6.8–10.6)	26.5 (23.4–29.5)
South	10.6 (8.7–12.5)	49.2 (46.2–52.2)	12.8 (10.6–14.9)	27.4 (24.7–30.1)
West	9.5 (7.2–11.7)	54.9 (51.0–58.7)	13.7 (10.9–16.6)	22.0 (18.8–25.1)
**Children <18 years living in household^d^**				
Yes	11.7 (9.5–13.8 **b**	55.2 (51.8–58.5)	9.7 (7.7–11.8)	23.4 (20.5–26.3)
No	11.0 (9.6–12.3)	49.6 (47.5–51.7)	12.4 (10.9–13.9)	27.1 (25.2–28.9)
**Cigarette smoking status^e^**				
Never smoker	10.0 (8.5–11.5) **b**	52.6 (50.2–55.0)	12.0 (10.4–13.6)	25.4 (23.3–27.5)
Current smoker	16.8 (13.2–20.3)	44.9 (39.9–49.8)	10.5 (7.1–13.8)	27.9 (23.5–32.3)
Former smoker	11.7 (9.6–13.8)	52.6 (49.3–55.8)	10.7 (8.6–12.8)	25.0 (22.2–27.8)
**EVP use^f^**				
Never user	9.1 (7.9–10.2) **b**	52.5 (50.5–54.4)	12.2 (10.9–13.6)	26.3 (24.6–28.0)
Current user	36.8 (28.7–45.0)	28.2 (20.8–35.5)	8.0 (3.4–12.7)	27.0 (19.1–34.8)
Former user	16.9 (12.9–20.9)	51.1 (45.4–56.8)	8.5 (5.4–11.7)	23.4 (18.7–28.2)

Abbreviation: CI, confidence interval.

a “Perception of harm” was categorized as “less harmful,” “equally harmful,” “more harmful,” or “don’t know” on the basis of responses to the question “Do you believe it is less harmful, equally harmful, or more harmful for pregnant women to use electronic vapor products than to smoke regular cigarettes?” Missing data were excluded from the analysis for cigarette smoking status (2.7%), EVP use status (0.5%), and whether a child under age 18 was living in the household (0.3%).

b Significant χ^2^ test (*P* < .05) indicated difference across groups within the specified characteristic.

c Northeast: Connecticut, Maine, Massachusetts, New Jersey, New Hampshire, New York, Pennsylvania, Rhode Island, and Vermont; Midwest: Illinois, Indiana, Iowa, Kansas, Michigan, Minnesota, Missouri, Nebraska, North Dakota, Ohio, South Dakota, and Wisconsin; South: Alabama, Arkansas, Delaware, District of Columbia, Florida, Georgia, Kentucky, Louisiana, Maryland, Mississippi, North Carolina, Oklahoma, South Carolina, Tennessee, Texas, Virginia, and West Virginia; West: Alaska, Arizona, California, Colorado, Hawaii, Idaho, Montana, Nevada, New Mexico, Oregon, Utah, Washington, and Wyoming.

d Defined as responding *yes* to having at least one child younger than 18 years living in the household.

e Current cigarette smokers were defined as respondents who reported smoking at least 100 cigarettes during their lifetime and who currently smoked cigarettes every day or some days. Former smokers were defined as respondents who reported having smoked at least 100 cigarettes in a lifetime, and who reported smoking not at all at the time of survey. Never cigarette smokers were defined as respondents who reported not having smoked 100 cigarettes in their lifetime.

f Current EVP users were defined as respondents who reported using any of the following products in their lifetime and within the previous 30 days: electronic cigarettes, such as Blu, 21st Century Smoke, or NJOY; electronic hookahs, hookah pens, or vape pens, such as Starbuzz or Fantasia; or some other electronic vapor product such as electronic cigars or electronic pipes. Former EVP users were defined as respondents who reported using EVPs at least once in their lifetime, and who reported not using EVPs in the past 30 days. Never EVP users were defined as respondents who reported not using EVPs in their lifetime and not using EVPs in the past 30 days.

Among reproductive aged women (18–44 y), 9.6% believed that EVP use by pregnant women was less harmful than conventional cigarette smoking, 59.2% believed it was equally harmful, 9.3% believed it was more harmful, and 21.8% reported “don’t know” **(** Table 2 **).** Among reproductive aged women, the highest percentage of respondents who perceived EVP use to be less harmful than cigarette smoking was observed among those with less than a high school diploma (12.5%) and current EVP users (31.5%).

**Table 2:** Perception of Harmful Effects of Electronic Vapor Products (EVPs) Versus Cigarette Smoking During Pregnancy^a^ Among Women Of Reproductive Age (18–44 y) and Women of Nonreproductive Age (≥45 y), by Sociodemographic Characteristics, *Styles* Survey, United States, 2015

Characteristic	Women of Reproductive Age (n = 820)				Women of Nonreproductive Age (n = 1,398)			
	Less Harmful, % (95% CI)	Equally Harmful, % (95% CI)	More Harmful, % (95% CI)	Don't Know, % (95% CI)	Less Harmful, % (95% CI)	Equally Harmful, % (95% CI)	More Harmful, % (95% CI)	Don't Know, % (95% CI)
**Overall**	9.6 (7.2–12.1)^b^	59.2 (55.3–63.2)	9.3 (6.9–11.7)	21.8 (18.5–25.2)	7.9 (6.2–9.5)	50.9 (47.9–53.9)	13.4 (11.2–15.6)	27.8 (25.1–30.5)
**Race/ethnicity **								
Non-Hispanic white	10.8 (7.7–13.9)^b^	59.6 (54.8–64.4)	6.2 (4.0–8.4)	23.4 (19.2–27.6)	8.4 (6.5–10.3)^c^	53.3 (50.0–56.5)	10.0 (7.9–12.1)	28.3 (25.4–31.3)
Non-Hispanic black	7.2 (0.5–14.0^b^	53.3 (41.2–65.5)	19.6 (9.8–29.4)	19.8 (10.4–29.3)	4.5 (1.1–7.8)	39.6 (30.1–49.1)	28.4 (19.3–37.4)	27.6 (18.9–36.3)
Hispanic	8.6 (2.3–14.8)	57.9 (47.8–68.0)	11.3 (4.6–17.9)	22.3 (13.8–30.8)	9.6 (2.1–17.1)	45.3 (34.5–56.2)	18.2 (9.9–26.4)	26.9 (17.0–36.8)
Non-Hispanic other	7.2 (0.1–14.3)	65.4 (51.3–79.4)	12.8 (2.3–23.3)	14.6 (4.5–24.7)	4.4 (0-10.9)	54.0 (36.9–71.2)	18.7 (4.6–32.7)	22.9 (7.8–38.0)
**Education **								
<High school diploma	12.5 (2.4–22.7)^c^	43.0 (28.4–57.5)	10.1 (2.1–18.0)	34.4 (20.3–48.6)	11.8 (3.9–19.7)^c^	32.4 (21.7–43.1)	18.8 (9.9–27.7)	37.0 (25.6–48.5)
High school graduate	6.6 (2.4–10.9)^b^	60.9 (52.2–69.6)	10.3 (4.0–16.6)	22.2 (15.2–29.1)	7.9 (5.0–10.8)	48.4 (43.4–53.3)	14.4 (10.9–18.0)	29.3 (24.7–33.9)
Some college	10.7 (6.2–15.2)^b^	59.1 (52.3–65.8)	12.9 (8.3–17.5)	17.3 (12.3–22.4)	7.8 (4.9–10.6)	53.8 (48.4–59.3)	12.5 (8.3–16.6)	25.9 (21.3–30.6)
College degree or more	9.5 (5.8–13.2)	63.5 (57.3–69.7)	5.1 (2.4–7.8)	21.9 (16.5–27.4)	6.2 (4.0–8.5)	59.1 (53.7–64.4)	10.8 (6.7–14.9)	23.9 (19.5–28.3)
**Annual household income, $ **								
<25,000	14.6 (3.5–25.8)^b^	50.0 (36.7–63.3)	11.0 (4.2–17.7)	24.4 (13.7–35.1)	10.2 (5.3–15.1)^c^	41.2 (33.6–48.7)	14.3 (8.8–19.8)	34.3 (26.7–41.9)
25,000–49,999	6.5 (3.6–9.4)^b^	59.6 (52.4–66.8)	9.5 (5.4–13.6)	24.3 (17.7–30.9)	7.1 (4.0–10.2)	45.6 (39.9–51.3)	19.3 (14.4–24.2)	28.0 (22.9–33.1)
50,000–99,999	7.6 (4.1–11.1)^b^	61.5 (55.1–67.8)	11.2 (6.7–15.6)	19.8 (14.6–24.9)	8.0 (5.2–10.8)	52.1 (47.0–57.2)	11.9 (8.2–15.7)	28.0 (23.5–32.5)
≥100,000	13.7 (8.0–19-4)	58.8 (50.5–67.1)	5.7 (1.4–10.0)	21.8 (14.8–28.8)	6.7 (3.7–9.7)	63.0 (57.0–69.0)	8.3 (4.5–12.1)	22.1 (17.0–27.1)
**Marital status **								
Married/living with partner	8.8 (6.0–11.6)^b^	58.6 (53.5–63.6)	10.3 (6.9–13.6)	22.4 (18.1–26.6)	7.5 (5.5–9.5)	52.0 (48.2–55-8)	12.5 (9.8–15.3)	27.9 (24.6–31.3)
Single	11.4 (6.6–16.1)^b^	62.0 (55.1–68.8)	6.8 (3.3–10.3)	19.9 (14.3–25.5)	10.9 (3.8–17.9)	44.5 (34.8–54.1)	16.6 (9.2–24.0)	28.1 (19.9–36.2)
Divorced/widowed/separated	5.9 (0-12.2)	46.3 (29.4–63.1)	17.4 (5.1–29.7)	30.4 (14.4–46.3)	7.6 (4.6–10.6)	50.7 (44.9–56.6)	14.3 (10.0–18.6)	27.3 (21.9–32.8)
**US census region^d^**								
Northeast	14.1 (6.7–21.6)^b^	55.6 (45.9–65.3)	6.7 (1.5–11.9)	23.6 (15.5–31.7)	9.9 (5.7–14.2)	48.4 (41.5–55.3)	10.5 (5.8–15.2)	31.2 (24.7–37.6)
Midwest	12.9 (7.1–18.7)^b^	60.8 (52.7–68.9)	6.0 (2.6–9.4)	20.3 (13.5–27.1)	7.3 (4.3–10.2)	52.7 (47.1–58.2)	12.1 (8.2–16.0)	28.0 (23.0–33.0)
South	8.2 (4.3–12.1)^b^	58.8 (52.1–65.6)	10.2 (6.0–14.4)	22.8 (16.9–28.6)	9.2 (6.2–12.3)	48.2 (43.1–53.2)	14.3 (10.4–18.1)	28.3 (23.9–32.9)
West	5.9 (2.3–9.3)	60.8 (52.8–68.8)	12.6 (6.9–18.3)	20.7 (14.27.3)	4.1 (1.4–6.7)	56.5 (49.3–63.7)	16.0 (10.4–21.7)	23.4 (17.1–29.7)
**Children <18 years living in household^e^**								
Yes	8.6 (5.7–11.6)	58.8 (53.5–64.1)	9.1 (5.9–12.2)	23.5 (18.8–28.1)	13.5 (7.4–19.6)^c^	48.0 (41.0–55.0)	14.1 (8.4–19.8)	24.4 (18.3–30.6)
No	10.7 (6.7–14.6)^b^	59.7 (53.7–65.7)	9.6 (5.9–13.3)	20.0 (15.2–24.9)	7.0 (5.4–8.7)	51.6 (48.2–54.8)	13.4 (10.9–15.8)	28.1 (25.1–31.0)
**Cigarette smoking status^f^**								
Never smoker	9.4 (6.5–12.4)^b^	61.4 (56.7–66.1)	8.5 (5.8–11.1)	20.7 (16.7–24.6)	5.0 (3.2–6.8)^c^	52.0 (47.8–56.1)	14.9 (11.7–18.0)	28.2 (24.4–31.9)
Current smoker	11.9 (4.7–19.1)	55.4 (43.4–67.4)	10.2 (1.7–18.7)	22.5 (13.3–31.8)	20.7 (13.3–28.1)	40.5 (32.0–49.0)	10.1 (3.9–16.3)	28.7 (21.3–36.1)
Former smoker	10.7 (4.5–16.8)	52.4 (42.3–62.5)	12.8 (5.4–20.3)	24.1 (15.3–32.8)	7.7 (5.0–10.5)	54.1 (48.9–59.4)	12.1 (8.5–15.7)	26.0 (21.4–30.6)
**EVP use^g^**								
Never user	8.7 (6.1–11.3)^b^^,^ c	60.0 (55.6–64.4)	9.9 (7.1–12.7)	21.3 (17.7–25.0)	5.5 (4.0–7.1)^c^	53.1 (49.9–56.4)	14.0 (11.6–16.4)	27.4 (24.5–30.3)
Current user	31.5 (13.7–49.3)	33.4 (16.3–50.5)	5.4 (0-15.5)	29.7 (12.7–46.8)	32.8 (19.8–30.4)	24.2 (11.7–36.7)	10.8 (0.3–21.3)	32.2 (17.8–46.5)
Former user	8.4 (2.6–14.3)	64.5 (54.3–74.7)	7.4 (2.9–12.0)	19.6 (10.9–28.3)	21.6 (12.8–30.4)	40.8 (30.1–51.4)	8.5 (1.2–15.9)	29.1 (20.0–38.3)

Abbreviation: CI, confidence interval.

a “Perception of harm” was categorized as “less harmful,” “equally harmful,” “more harmful,” or “don’t know” on the basis of responses to the question “Do you believe it is less harmful, equally harmful, or more harmful for pregnant women to use electronic vapor products than to smoke regular cigarettes?” A total of 30 cases (3.6%) were excluded from the multivariate model due missing data for at least one of the assessed variables.

b Significant χ^2^ test (*P* < .05) indicated difference across groups within the specified characteristic

c Significant test of difference in proportions (*P* < .05) between women of reproductive age and women of non-reproductive age for each characteristic.

d Northeast: Connecticut, Maine, Massachusetts, New Jersey, New Hampshire, New York, Pennsylvania, Rhode Island, and Vermont; Midwest: Illinois, Indiana, Iowa, Kansas, Michigan, Minnesota, Missouri, Nebraska, North Dakota, Ohio, South Dakota, and Wisconsin; South: Alabama, Arkansas, Delaware, District of Columbia, Florida, Georgia, Kentucky, Louisiana, Maryland, Mississippi, North Carolina, Oklahoma, South Carolina, Tennessee, Texas, Virginia, and West Virginia; West: Alaska, Arizona, California, Colorado, Hawaii, Idaho, Montana, Nevada, New Mexico, Oregon, Utah, Washington, and Wyoming.

e Defined as responding *yes* to having at least one child less than 18 years of age living in the household.

f Current cigarette smokers were defined as respondents who reported smoking at least 100 cigarettes during their lifetime and who currently smoked cigarettes “every day” or “some days”. Former smokers were defined as respondents who reported having smoked at least 100 cigarettes in a lifetime, and who reported smoking “not at all” at the time of survey. Never cigarette smokers were defined as respondents who reported not having smoked 100 cigarettes in their lifetime.

g Current EVP users were defined as respondents who reported having used any of the following products within the past 30 days: electronic cigarettes, such as Blu, 21st Century Smoke, or NJOY; electronic hookahs, hookah pens, or vape pens, such as Starbuzz or Fantasia; or some other electronic vapor product such as electronic cigars or electronic pipes. Former EVP users were defined as respondents who reported using EVPs at least once but who reported not using EVPs in the previous 30 days. Never EVP users were defined as respondents who reported never having used EVPs.

Among nonreproductive aged women (≥45 y), 7.9% believed that EVP use by pregnant women was less harmful than conventional cigarette smoking, 50.9% believed it was equally harmful, 13.4% believed it was more harmful, and 27.8% reported “don’t know.” The highest percentage of respondents who perceived EVP use to be less harmful than cigarette smoking was observed among Hispanics, those with less than a high school diploma, those with annual household income less than $25,000, those with children younger than 18 years living in the household, current cigarette smokers, and current EVP users.

Harm perceptions differed between women of reproductive and nonreproductive age, overall and by selected race/ethnic groups, educational and income levels, marital status, US Census regions, whether a child younger than 18 years lived in the household, cigarette smoking status, and EVP use (Table 2) (*P* < .05). Compared with nonreproductive-aged women, the percentage of reproductive-aged women who perceived EVP use during pregnancy to be less harmful than cigarette smoking was higher among non-Hispanic whites, non-Hispanic blacks, those with some college education, those with an annual household income less than $25,000, those single or married, those living in the Northeast or Midwest, those without a child aged younger than 18 years living in the household, never cigarette smokers, and never EVP users (*P* < .05). In contrast, compared with nonreproductive aged women, the percentage of reproductive aged women who perceived EVP use to be less harmful than cigarette smoking during pregnancy was lower among high school graduates, those with an annual household income of $25,000 to $99,999, and those living in the South (*P* < .05).

## Discussion

These findings indicate that US adults have varying perceptions about the harms of EVP use versus cigarette smoking during pregnancy. About half of adults studied perceived EVP use to be as harmful as smoking, whereas over one-quarter reported they did not know. Variations in harm perceptions were observed among reproductive aged women (9.6%) and nonreproductive aged women (7.9%). Perception of relative harm also differed by sociodemographic factors and current tobacco use. Among all adults and among women of all ages, a higher percentage of current EVP users than never users believed EVP use was less harmful than cigarette smoking. These results suggest that public health educational efforts may be warranted to inform the public about the health risks of using all forms of tobacco during pregnancy, including EVPs. In addition to public health messaging, clinicians and other health care providers should ask all women of reproductive age whether they are using any tobacco products, including EVPs, and advise them of the harmful effects of using these products during pregnancy.

EVPs are perceived as safer than cigarettes, especially during high-risk conditions such as pregnancy (12–16). A small study of pregnant women who attended a university’s outpatient clinic found that 43% of participants believed that EVPs were less harmful to a fetus than conventional cigarettes, and 57% believed that EVPs contained nicotine (18). Among ever users of EVPs, 74% believed that EVPs were less harmful than conventional cigarettes (18). This perception, which could be partly due to EVP advertising (19,20), could lead reproductive aged women who do not smoke to be open to using EVPs and pregnant women who smoke to switch to EVPs or use them to reduce cigarette smoking instead of quitting tobacco use entirely (15).

Although current research indicates that EVP aerosol has fewer harmful constituents than conventional cigarette smoke, EVP use during pregnancy is not risk-free (3,5–7). One concern with EVP use during pregnancy, with or without concurrent cigarette smoking, is that many EVP products contain nicotine (3,7). The US Surgeon General has concluded that evidence is sufficient to warn pregnant women and those of reproductive age about the harmful effects of using any nicotine products during pregnancy (1). 

This study has limitations. First, although *Styles* uses an address-based probability sample of the US population, certain populations are less likely to respond. However, *Styles* data are weighted to be nationally representative, and tobacco use estimates from *Styles* are consistent with other national household surveys (21). Second, because of the questions on the *Styles* survey, former EVP use was ascertained by using ever use as a threshold for regular use. Given that it was not possible to distinguish between former EVP users who routinely used the products and those who only briefly experimented with the products, this classification could introduce bias. Third, the sensitive nature of the questions may have led some participants to provide more socially acceptable responses rather than provide their true perception. However, given that the survey was completed on the Internet, the likelihood of such bias is probably minimal (22).

This study found that a higher percentage of reproductive aged women than nonreproductive aged women believed EVP use was less harmful than cigarette smoking, overall and by sociodemographic characteristics and tobacco use status. A higher percentage of EVP users than never EVP users believed EVP use was less harmful than conventional cigarette smoking. These findings have several implications for public health policy, planning, and practice. First, when addressing potential public health harms associated with EVP use, proven strategies should be implemented to prevent and reduce all forms of tobacco use, including tobacco price increases, comprehensive smoke-free laws, high-impact media campaigns, barrier-free cessation treatment and services, and comprehensive statewide tobacco control programs (1). A comprehensive approach can reduce the use of all tobacco products, including by pregnant women. Second, clinicians and other health care providers should ask all women of reproductive age whether they are using any tobacco products, including EVPs, and advise them about the harmful effects of using products that contain nicotine and other toxicants during pregnancy. Finally, efforts are warranted 1) to educate the public, particularly cigarette smokers and EVP users, about the health risks of EVP use during pregnancy, 2) to provide advice and assistance to those who want to quit, and 3) to promote quitting all forms of tobacco use.
